# Genetic admixture and cardiovascular disease risk in postmenopausal Hispanic women^☆^

**DOI:** 10.1016/j.ijcard.2022.08.020

**Published:** 2022-08-10

**Authors:** Monica D. Zuercher, Danielle J. Harvey, Lauren E. Au, Aladdin H. Shadyab, Rami Nassir, John A. Robbins, Michael F. Seldin, Lorena Garcia

**Affiliations:** a University of California Davis, Davis, CA, United States; b Herbert Wertheim School of Public Health and Human Longevity Science, University of California San Diego, La Jolla, CA, United States; c Department of Pathology, School of Medicine, Umm Al-Quraa University, Saudi Arabia

**Keywords:** Genetics, Stroke, Coronary heart disease, Latinos

## Abstract

**Background:**

Hispanics are a heterogeneous population with differences in the prevalence of cardiovascular disease (CVD) and its related risk factors among ethnic sub-groups. This study evaluated the association of genetic admixture and CVD in self-identified Hispanic women from the Women’s Health Initiative (WHI).

**Methods:**

Data came from the WHI Observational Study and the Clinical Trial Components conducted among postmenopausal women. The CVD outcomes included coronary heart disease (CHD) and stroke. The proportions of European (EUR), sub-Saharan African (AFR), and Amerindian (AMI) admixture were estimated using 92 ancestry-informative markers. Cox regression models were used to assess the relationship between genetic admixture and CVD adjusting for age, lifestyle risk factors, known risk factors, and neighborhood socioeconomic status.

**Results:**

Among 5195 participants EUR ancestry was associated with a lower CHD risk after adjusting for age (HR 0.41, *p* = 0.02), and in the fully adjusted model (HR 0.40, *p* = 0.03). AFR ancestry was associated with a higher CHD risk after adjusting for age (HR 2.91, p = 0.03), but it only showed a trend in in the fully adjusted model (HR 2.46, *p* = 0.10). AMI ancestry was not statistically significantly associated with CHD and none of the genetic admixture proportions were statistically significantly associated with stroke (*p* > 0.05).

**Conclusion:**

EUR ancestry was associated with a lower risk of CHD in Hispanic women. This highlights the need to account for genetic admixture in future CVD studies to consider different heritage groups to understand the role that genetic, neighborhood socioeconomic status, and environmental factors contribute to CVD health disparities in Hispanic women.

Cardiovascular disease (CVD) is the leading cause of death and disability in the United and is the leading cause of death among Hispanics in the U.S. [1,2]. Hispanics are a diverse population that varies in national origin, immigration status, socioeconomic characteristics, genetic backgrounds, and cultural histories [3,4]. The Hispanic mortality paradox refers to the finding that Hispanics have lower mortality rates than non-Hispanic White Americans despite having lower levels of income, education and access to health care, and a higher risk of obesity and diabetes than non-Hispanic White Americans [3-5]. The combination of genetic, neighborhood socioeconomic status and environmental factors contribute to the differences in the CVD risk observed between populations. Despite this, there is a lack of health data for Hispanic populations, especially in the area of CVD and its risk factors [5]. Genetic admixture uses ancestry informative markers to identify the ancestral genetic background of an individual [6,7]. Hispanics are an admixed group with varying proportions of ancestral European (EUR), Amerindian (AMI) and sub-Saharan African (AFR) ancestry contributions as the result of multiple factors and historical events like population movements, colonization, and enslavement, among others [8,9]. To shed greater light on the link between genetic admixture and CVD risk in Hispanic populations, this study evaluated the association between the relative proportion of AFR, AMI and EUR admixtures and CVD risk in a subset of Women’s Health Initiative Hispanic participants with genotyping data. The amount of variation in CVD risk explained by genetic components, neighborhood socioeconomic status, known CVD risk factors, and lifestyle behaviors was also evaluated.

## 1. Methods

The Women’s Health Initiative (WHI) is one of the most important large health studies focusing on women’s health that enrolled 161,808 postmenopausal women across 40 WHI clinical centers nationwide between October 1, 1993, and December 31, 1998. Participants of the WHI study ranged in age from 50 to 79 years and were either randomized into one of the three clinical trials (Hormone Therapy (HT) Trial, the Diet Modification (DM) Trial, and the Calcium and Vitamin D (CaD) Trial) or enrolled into an observational study (OS) [10]. All women provided written informed consent, and the study was approved by the local institutional review boards as well as by the Coordinating Center Institutional Review Board and the National Institutes of Health [11]. The current study included 5195 postmenopausal women who self-identified as Hispanic (2927 from the observational study and 2268 from the clinical trials). Participants with a history of CHD and/or stroke events at baseline (*N* = 80) and for whom DNA samples were not successfully genotyped (*N* = 1289) were excluded.

This study focused on incident CHD and stroke based on self-report of physician diagnosis and time-to-CHD/stroke occurrence were the outcomes of interest. CHD was defined as hospitalized myocardial infarction, definite silent myocardial infarction, or death due to coronary disease. Stroke was defined as rapid onset of a persistent neurologic deficit attributed to an obstruction or rupture of the brain arterial system, lasting >24 h and without evidence for other cause. Only strokes requiring hospitalization were considered outcome events for WHI [12]. The CVD outcomes were adjudicated and ascertained by physician review of medical records, as previously described [12].

Genetic admixture was calculated using a marker set of 92 ancestry informative markers that demonstrated large differences in allele frequency between ancestral populations from Europe, sub-Saharan Africa, and the Americas (>45%) [6,13]. These SNPs demonstrated reproducible genotyping results in population samples of diverse origin, had >90% complete typing results in each population, were in Hardy-Weinberg equilibrium (*p* > 0.005) in parental populations and this set of AIMs was validated to differentiate parental populations [13].

Genotyping was performed using the TaqMan OpenArrays system (Life Technologies/Applied Biosystems, Foster City, CA, USA) [6]. Genotypes were scored using the OpenArray SNP Genotyping Analysis Software provided by the manufacturer. All ancestry informative marker (AIM) single-nucleotide polymorphisms had >93% call rate and showed >98% concordance in 5% duplicate assays [6].

Admixture proportions were determined using the Bayesian clustering algorithms implemented in the program STRUCTURE v2.1. Each analysis was performed without any prior population assignment and was performed at least 3 times with similar results using >200,000 replicates and > 100,000 burn-in cycles under the admixture model. The analyses were performed using representatives of the three parental populations (96 samples each) and under the assumption of three populations (K = 3; AFR, AMI and EUR). The analysis for the subset of individuals with >10% AMI and < 5% AFR ancestry (2018 of the 5195 self-identified Hispanics) was performed with representatives of the two parental populations (K = 2; AMI and EUR) under the assumption of two populations [14].

The study covariates included neighborhood socioeconomic status, known CVD risk factors, and lifestyle behaviors. These variables were included because they are mediators of the association between genetic admixture and CVD so we included them in the models as covariates to have a measure of the direct effect of genetic admixture over CVD risk that is not explained by those mediators. The study covariates were determined using baseline data from both the observational study (OS) and clinical trial (CT) components of the WHI study. Neighborhood socioeconomic status (NSES) was evaluated with a composite variable. The NSES was obtained using a standardized geocoding protocol, which linked individual WHI participant addresses to the year 2000 U.S. Census Federal Information Processing Standards (FIPS) codes and tract-level socioeconomic data. A summary measure of each participant’s neighborhood socioeconomic environment was estimated from the tract-level data using six variables representing several dimensions of wealth and income [15].

Health-related variables were also included: physical activity, smoking status, alcohol intake, diet quality, body mass index (BMI), and other chronic diseases. Physical activity was evaluated with a validated physical activity questionnaire, and it was included in the model as the total minutes of recreational physical activity per week, including walking, mild, moderate, and strenuous physical activity [16]. Smoking status was determined with information from the personal habits’ questionnaire, and this combined questions into a dichotomous smoking status variable (never or past/current smoker). Alcohol intake in grams per day was estimated using a food frequency questionnaire [17]. Diet quality was assessed using the Healthy Eating Index (HEI)-2015 [18]. For BMI, weight was measured to the nearest 0.1 kg on a balance beam scale. Height was measured to the nearest 0.1 cm using a wall-mounted Harpenden stadiometer. BMI was calculated as weight (kg) divided by the square of measured height (m^2^) [19]. Hypertension was defined as systolic pressure ≥ 140 mmHg or diastolic ≥90 mmHg or self-reported hypertension with the use of antihypertensive medication [20]. Diagnosis of diabetes at baseline was obtained from the medical history questionnaire in response to the question “Did a doctor ever say that you had sugar diabetes or high blood sugar when you were not pregnant?” [21]. Hypercholesterolemia was defined by self-report at baseline and then by use of lipid-modulating medication [20].

Two-sample *t*-tests were applied to compare the mean differences of each continuous variable between participants with and without CVD. Chi-square tests were used to compare the two groups on categorical variables. Separate Cox regression models were fit to examine the association between admixture proportion and CHD/stroke. The analyses were restricted only to follow-up events. CHD and stroke status were used as dichotomous traits (0 = no, 1 = yes) as the indicator variable for failure/censorship. The survival time for participants who did not develop the CVD outcome of interest was defined as the days from enrollment to the end of follow-up (the follow-up time for CVD events in this analysis includes data until September 2018). Hazard ratios (HRs) and 95% confidence intervals (CI’s) are presented for each model. African, European, and AMI admixtures proportions were examined in separate models. A unit increase in admixture was defined as the effect of the 100% admixture compared with no admixture of the specific ancestry population. Comparing the effects of 100% vs. 0% admixture corresponds to comparing one parental population to another parental population. Cox regression models were fitted with and without adjusting for age at entry, lifestyle-related risk factors (smoking, alcohol intake, diet quality, and physical activity), known CVD risk factors (diabetes, hypertension, hypercholesterolemia, and body mass index), and neighborhood socioeconomic status (NSES). These covariates were serially added to the model and the final model included all covariates. The amount of variation explained by the genetic admixture variables and the different sets of covariates in the Cox regression models was calculated using the coefficient of explained randomness proposed by O’Quigley et al. [22] Finally, multiple imputation with the fully conditional specification method was used to estimate missing values of the variable neighborhood socioeconomic status (*N* = 370 which represents 7.1% of the observations) and physical activity (*N* = 253 which represents 4.9% of the observations) assuming that data were missing at random. Analyses were performed using SAS software version 9.4 (SAS Institute, Cary, NC USA). All statistical tests were two-sided and *p* ≤ 0.05 was considered statistically significant.

## 2. Results

In the overall sample of Hispanic women, 50.35% were Mexican, 11.66% were Puerto Rican, 7.69% were Cuban, and 25.31% self-identified as other Hispanic/Latina. The average ancestry proportions were 13.6% AFR, 59.7% EUR and 26.7% AMI. The incidence of cardiovascular disease was 3.43% for coronary heart disease and 2.83% for stroke during a median follow-up of 12.9 years (range, 0.1–24.0 years).

Women of Puerto Rican origin had the highest incidence of CHD (3.9%), followed by Mexican women (3.7%), and women in the category of other Hispanic/Latina (3.1%); Cuban women had the lowest CHD incidence of all the Hispanic heritage-groups (1.6%). Women of Puerto Rican origin had the highest incidence of stroke (4.5%), followed by Cuban women (3.6%), and women in the category of other Hispanic/Latina (3.0%); Mexican women had the lowest incidence of stroke of all the Hispanic heritage-groups (2.5%).

Women with a higher EUR ancestry were significantly older at baseline, had higher values of neighborhood SES, physical activity, alcohol intake and diet quality, and were more likely to have incident stroke and to smoke than women with a lower European ancestry (*p* < 0.05) (Table 1). Women with a higher European ancestry also had lower BMI and were less likely to use English as their preferred language, to have family history of diabetes and to be allocated in the hormone treatment arm than their counterparts with lower EUR ancestry (*p* < 0.05). There were no statistically significant differences in the incidence of CHD and prevalence of hypertension and hypercholesterolemia between women with higher and lower EUR ancestry (*p* > 0.05).

EUR ancestry was associated with a lower risk of CHD when adjusting for age at baseline (HR 0.41, *p* = 0.02) and this association remained after additional adjustment by covariates (HR 0.40, *p* = 0.03) (Table 2). AFR ancestry was associated with a higher risk of CHD when adjusting for age at baseline (HR 2.91, p = 0.03) and lifestyle-related covariates (HR 2.89, *p* = 0.04). However, when known risk factors and NSES were added to the model the analysis only showed a trend (*p* = 0.07 and *p* = 0.10, respectively). AMI ancestry was not associated with CHD in any of the models (*p* > 0.2). None of the genetic ancestry proportions were found to be statistically significantly associated with stroke in any of the models (p > 0.2) (Table 3).

The variance of the risk of CHD explained by the genetic admixture variables was low (Table 4). AMI ancestry only explained 0.25% of the variance of the risk, AFR explained 1.76% and EUR ancestry 1.92%. From the different sets of covariates, most of the variance of the risk of CHD was explained by age, known risk factors, lifestyle-related risk factors and NSES, in that order. The fully adjusted models using the different genetic ancestries explained between 43 and 44% of the variation in CHD risk.

The same pattern was observed with stroke where the genetic admixture variables explained a low proportion of the variation of the risk in stroke (AMI 1.86%, AFR 0.37% and EUR 0.85%), and most of the variation was explained by the covariates. The fully adjusted models using the different genetic ancestries explained ~43% of the variation in the risk of stroke.

## 3. Discussion

Because of the high prevalence of CVD in Non-Hispanic White Americans and Black Americans in comparison with the low incidence of CVD in Hispanics, we hypothesized that for Hispanic women EUR and AFR ancestries would be associated with higher risks of CVD and that AMI ancestry would be associated with lower risk. Contrary to what we hypothesized, EUR ancestry was associated with a lower risk of CHD and in concordance with our hypotheses, AFR ancestry was associated with a higher risk of CHD.

Our results contradict the results found in the Multi-Ethnic Study of Atherosclerosis (MESA) study where EUR ancestry was associated with a higher prevalence of coronary artery calcium, a preclinical indicator of CHD [23]. The MESA examined the association of genetic admixture with coronary artery calcium (CAC) and common and internal carotid intima-media thickness, two indicators of subclinical CVD among 705 Hispanics [23]. Researchers found that the highest tertile of European ancestry was associated with a 34% higher CAC prevalence (*p* = 0.02) when compared with the lowest tertile, and Amerindian (AMI) ancestry was not associated with CAC (prevalence OR = 0.99; 95% CI, 0.92–1.07) [ 23]. Some of these inconsistencies with our results could be explained by differences that need to be considered such as the different study populations (the MESA study included both men and women in a wider range of age at enrollment than the WHI (aged 45–84 years in MESA vs. 50–79 years in WHI) or that EUR ancestry is associated with a higher risk of subclinical CVD conditions but is not enough to trigger clinical conditions.

In our study, AFR ancestry was associated with a higher risk of CHD, but after including known risk factors and NSES to the models the association only showed a trend. This finding contrasts with the results of the Boston Puerto Rican Health Study where AFR ancestry was associated with a lower prevalence of CVD [24]. Results from the Boston Puerto Rican Health Study showed that in 1129 self-identified Puerto Ricans living in the greater Boston metropolitan area, sub-Saharan African (AFR) ancestry was associated with lower odds of CVD (OR = 0.32, 95% CI 0.10–1.00) [24]. The differences in the findings could be explained by differences in the characteristics of the participants of the studies. The Boston Puerto Rican Health Study included both men and women, also the proportion of AFR ancestry in people of Puerto Rican heritage is higher than that of the other Hispanic heritage groups included in the WHI so the contribution of the AFR ancestry to the development of CVD could differ within Hispanic heritage-groups [24]. Regarding the effect of AFR ancestry over the risk of CVD, it has been previously reported that a substantial proportion of the effect of AFR ancestry over the risk of disease can be attributed to socioeconomic and cultural factors like barriers to access to health care, racism, and discrimination, among others [14,25]. A study that involved 87 adults from Puerto Rico evaluated if African ancestry or a social classification of skin pigmentation (*color*: blanco or white, the intermediate category trigueño, and negro or black) better predicts blood pressure. Results showed that skin *color* better predicts blood pressure than does a genetic-based estimate of continental ancestry so the authors concluded that associations between genetic ancestry and health may be attributable to sociocultural factors related to race and racism, rather than to functional genetic differences between racially defined groups [26]. Thus, we cannot discard the possibility of residual confounders affecting the association between AFR ancestry and CVD in our study, especially because the fully adjusted models with using the three genetic ancestries only explained between 43% and 44% of the variation of the risk of CVD which means that >55% of the variation in the risk can be attributed to unmeasured social and environmental factors.

In our study, AMI ancestry was not found to be statistically significantly associated with any of the CVD outcomes analyzed. Another study found no association between AMI ancestry and CVD [23]. In contrast, the Boston Puerto Rican Health Study found that AMI ancestry was associated with a higher prevalence of CVD (OR = 16.63, 95% CI 1.34–211.20) [24]. The differences in the findings could be explained by differences in the characteristics of the participants of the studies (as was previously mentioned) and to differences in the definition of CVD used. In the Boston Puerto Rican Health Study CVD was defined as a positive response to the question “Have you ever been told by a physician that you have heart disease” or to similar questions on heart attack or stroke [24]. That CVD definition that incorporates information about multiple outcomes differs from the one in this study where separate models were fit for each CVD outcome. Additionally, the degree of accuracy in the classification of participants as having CVD or not having CVD differs between studies. In the WHI the CVD outcomes were adjudicated and ascertained, but in the Boston Puerto Rican Health Study CVD was evaluated by self-report only. It has been recognized that the diagnosis and treatment of CVD in Hispanics can be affected by multiple factors like limited access to health care, lower economic status, being born outside of the United States, lower education, and language or cultural barriers [27-29]. These factors can lead to misclassification of participants that have undiagnosed CVD or that reported not having CVD when indeed they have it.

Our findings suggest that the incidence of CVD in Hispanic women was driven more by modifiable factors than genetic admixture. Even if the contribution of the genetic admixture variables that explain the variance on the risk of CVD is low, we need to keep in mind that genetic admixture variables can affect other known CVD risk factors [30]. It has been reported that genetic admixture variables are associated with multiple CVD risk factors like diabetes, hypertension, and indicators of adiposity in Hispanic postmenopausal women so, by adjusting the models for these conditions, we could underestimate the overall effects of ancestry over the risk of CVD [14,15,31].

Known risk factors for cardiovascular disease explained most of the variation in the risk of CVD. This could be due to the multiple changes in hormone levels and body composition that are associated with menopause and which in turn have an impact on the development of CVD [32]. It has been reported that risk factors for hypertension can differ by gender and by age-group [33]. In this study, we found that major risk factors for CVD like hypertension and diabetes that are the result of long-term exposures or behaviors have a higher effect on the risk of CVD than more short-term risk factors like physical activity. Lifestyle-related risk factors explained a good amount of the variation in the risk of CVD. The type and intensity of physical activity that older adults perform may be lower than the level and intensity of the physical activity performed by younger adults, so it is reasonable to expect different impacts on the risk of CVD [34]. We also need to consider characteristics of the WHI participants such as the high educational level which can influence health behaviors or exclusion criteria such as alcoholism so the effect of alcohol consumption or other lifestyle-related factors on the risk of CVD in a population less educated or that includes heavy drinkers might be different from those observed in this study [10,11]. Finally, neighborhood socioeconomic status was the covariate that explained the least variation on the risk of CVD. The addition of neighborhood socioeconomic status (NSES) was commonly associated with reductions of the magnitude of the HRs and/or reduction of the statistical significance of the genetic admixture variables. This has been reported in previous studies and has been attributed to the role that socioeconomic status play as a mediator of the association between genetic ancestry and disease risk [15,35]. NSES has been associated with multiple CVD risk factors like access to healthy foods and/or safe places to be physically active, higher prevalence of hypertension, weight gain, and metabolic syndrome, availability of medical care, and psychological factors like stress [36,37]. This means that, just like with genetic admixture, the effect of NSES over the risk of CVD may be underestimated due to the adjustment for known and lifestyle-related risk factors in the model.

The strengths of this study include the prospective design and a large sample of Hispanic women with diverse ethnicity. In addition, CHD and stroke were adjudicated, and we had information on a robust set of confounders. Limitations included the low incidence of CVD, limiting power to detect small associations between the genetic admixture components and CVD, or to run analysis in subgroups of the sample. Other limitations include the small sample size for some Hispanic heritage groups that did not allow us to evaluate the relationship between genetic admixture and CVD within each Hispanic subgroup and the lack of information about psychosocial factors like racism, discrimination or social marginalization that could affect the risk of CVD.

In conclusion, EUR ancestry was associated with a lower risk of CHD while AFR showed a trend to increase the risk of CHD. AMI ancestry was not statistically significantly associated with any of the CVD outcomes after adjusting for covariates. It is hard to determine the contribution of genetic admixture to the development of CVD without recognizing the complex interaction between itself and the multiple factors that participate in the pathologic CVD process. Sociocultural factors need to be considered when analyzing the association between genetic ancestry and health outcomes. More studies need to be conducted to determine the role that genetic admixture plays in the development of CVD and whether associations differ by CVD outcome. Studies examining large populations of Hispanics should consider different heritage groups to have a better understanding of the factors that contribute to health disparities. Moreover, known CVD risk factors and lifestyle-related risk factors play an important role in the pathologic pathway between genetics and CVD development so these factors need to be considered to design more effective interventions to reduce the health disparities in Hispanic populations.

## Figures and Tables

**Table 1:** Descriptive characteristics between postmenopausal women with lower vs. higher proportion of European ancestry from the Women’s Health Initiative.

Variable	Tertile 1 (*n* = 1727)		Tertile 3 (*n* = 1731)		*P*-Value
	Mean	SD	Mean	SD	
Age (years)	59.6	6.8	60.8	6.8	<0.0001
Neighborhood SES (score)	66.7	10.6	70.4	10.4	<0.0001
Physical Activity (hr/wk)	147.4	169.9	166.8	186.8	0.002
Alcohol intake (g/day)	1.1	4.3	1.5	4.0	0.01
Diet quality (HEI-2015 score)	61.0	10.0	63.1	10.4	<0.0001
BMI (kg/m^2^)	29.3	5.7	28.6	5.9	0.0005
Variable	n	%	n	%	P-Value
Incident CHD* (yes)	56	3.2	54	3.1	0.84
Incident stroke* (yes)	40	2.3	60	3.5	0.04
Preferred language (English)	1319	76.4	1265	73.1	0.03
Smoking (yes)	570	33.5	653	38.7	0.002
Hypertension (yes)	654	37.9	652	37.7	0.90
Hypercholesterolemia (yes)	256	16.1	269	16.7	0.66
Diabetes (yes)	192	11.1	111	6.4	<0.0001
Hormone treatment arm (yes)	462	26.8	387	22.4	0.003

SES: socioeconomic status, HEI: Healthy Eating Index, BMI: body mass index, CHD: coronary heart disease.

* Incident CHD/stroke: women that develop CHD/stroke during the follow-up period. Women with a history of CHD and/or stroke at baseline were excluded.

**Table 2:** Association of genetic admixture and coronary heart disease in Hispanic postmenopausal women from the Women’s Health Initiative.

Admixture	Model	Variables	Hazard Ratio	95% CI	P- Value
EUR	1	Model 1	0.41	0.19, 0.89	0.02
	2	Model 1 + lifestyle-related risk factors	0.38	0.17, 0.83	0.01
	3	Model 2 + known risk factors	0.39	0.18, 0.88	0.02
	4	Model 3 + NSES	0.40	0.18, 0.91	0.03
AMI	1	Model 1	1.46	0.66, 3.21	0.35
	2	Model 1 + lifestyle-related risk factors	1.58	0.70, 3.56	0.27
	3	Model 2 + known risk factors	1.61	0.70, 3.69	0.26
	4	Model 3+ NSES	1.59	0.69, 3.65	0.27
AFR	1	Model 1	2.91	1.10, 7.67	0.03
	2	Model 1 + lifestyle-related risk factors	2.89	1.08, 7.69	0.04
	3	Model 2 + known risk factors	2.60	0.92, 7.35	0.07
	4	Model 3+ NSES	2.46	0.85, 7.10	0.10

EUR: European, AMI: Amerindian, AFR: Sub-Saharan African, NSES: neighborhood socioeconomic status.

Model 1: with age at baseline.

Model 2: with age at baseline and lifestyle-related risk factors.

Model 3: with age at baseline, lifestyle-related risk factors and known risk factors.

Model 4: with age at baseline, lifestyle-related risk factors, known risk factors and NSES.

Lifestyle-related risk factors: smoking, alcohol intake, diet quality and physical activity. Known risk factors: diabetes, hypertension, hypercholesterolemia, and body mass index.

Sample size 5047 women.

**Table 3:** Association of genetic admixture and stroke in Hispanic postmenopausal women from the Women’s Health Initiative.

Admixture	Model	Variables	Hazard Ratio	95% CI	P- Value
EUR	1	Model 1	1.19	0.50, 2.86	0.69
	2	Model 1 + lifestyle-related risk factors	1.12	0.46, 2.74	0.80
	3	Model 2 + known risk factors	1.40	0.55, 3.61	0.48
	4	Model 3 + NSES	1.45	0.56, 3.74	0.45
AMI	1	Model 1	0.63	0.25, 1.59	0.33
	2	Model 1 + lifestyle-related risk factors	0.66	0.26, 1.68	0.38
	3	Model 2 + known risk factors	0.63	0.23, 1.67	0.35
	4	Model 3+ NSES	0.62	0.23, 1.66	0.35
AFR	1	Model 1	1.72	0.50, 5.92	0.39
	2	Model 1 + lifestyle-related risk factors	1.82	0.52, 6.40	0.35
	3	Model 2 + known risk factors	1.25	0.31, 5.10	0.75
	4	Model 3+ NSES	1.20	0.29, 4.98	0.80

EUR: European, AMI: Amerindian, AFR: Sub-Saharan African, NSES: neighborhood socioeconomic status.

Model 1: with age at baseline.

Model 2: with age at baseline and lifestyle-related risk factors.

Model 3: with age at baseline, lifestyle-related risk factors and known risk factors.

Model 4: with age at baseline, lifestyle-related risk factors, known risk factors and NSES.

Lifestyle-related risk factors: smoking, alcohol intake, diet quality and physical activity. Known risk factors: diabetes, hypertension, hypercholesterolemia, and body mass index.

Sample size 4887 women.

**Table 4:** Percentage of variance in cardiovascular disease risk explained by the predictors.

Variables	% Variance: CHD	% Variance: Stroke
Amerindian Ancestry	0.25	1.86
Sub-Saharan African Ancestry	1.76	0.37
European Ancestry	1.92	0.85
Age	24.34	31.95
Neighborhood SES	1.35	1.17
Diabetes	15.04	11.94
Hypertension	19.93	9.95
Hypercholesterolemia	1.41	4.04
Body mass index	5.62	0.45
Smoking	0.12	0.02
Alcohol intake	7.33	2.48
Diet quality	0.02	0.38
Physical activity	1.66	0.43
Fully adjusted model AMI	43.26	43.34
Fully adjusted model AFR	43.27	43.35
Fully adjusted model EUR	44.12	43.08

SES: socioeconomic status, EUR: European, AMI: Amerindian, AFR: Sub-Saharan African.
